# Molecular Analysis of Retrogradation of Corn Starches

**DOI:** 10.3390/polym11111764

**Published:** 2019-10-27

**Authors:** Marek Sikora, Magdalena Krystyjan, Anna Dobosz, Piotr Tomasik, Katarzyna Walkowiak, Łukasz Masewicz, Przemysław Łukasz Kowalczewski, Hanna Maria Baranowska

**Affiliations:** 1 Department of Carbohydrates Technology, University of Agriculture in Kraków, 122 Balicka Street, 30-149 Kraków, Poland; rrsikora@cyf-kr.edu.pl (M.S.); magdalena.krystyjan@urk.edu.pl (M.K.); anna.dobosz88@gmail.com (A.D.); 2 Nantes Nano Nanotechnological Systems, 21 Dolne Młyny Street, 50-700 Bolesławiec, Poland; rrtomasi@cyf-kr.edu.pl; 3 Department of Physics and Biophysics, Poznań University of Life Sciences, 38/42 Wojska Polskiego Street, 60-637 Poznań, Poland; katarzyna.walkowiak@up.poznan.pl (K.W.); lukasz.masewicz@up.poznan.pl (Ł.M.); 4 Institute of Food Technology of Plant Origin, Poznań University of Life Sciences, 31 Wojska Polskiego Street, 60-624 Poznań, Poland; przemyslaw.kowalczewski@up.poznan.pl

**Keywords:** amylose, amylopectin, plant gums, relaxometry, spin–lattice relaxation times, spin–spin relaxation times

## Abstract

Changes of the molecular dynamics of water in 5% corn starch pastes and 5% systems composed of starch and non-starchy hydrocolloid were studied during short and long term retrogradation. Low Field NMR was used to record mean correlation times (τ_c_) of water molecules. This molecular parameter reflects the rotation of water molecules within the network of paste. Starches of different amylose and amylopectin content were selected for this study. Comparison of the changes of τ_c_ shows how particular polymers bind water molecules. During 90 days of storage, over 50% increase in mean correlation time was recorded in pastes of starches with high amylose content. This suggests that the formation of polymeric network is controlled by amylose to which water is binding. Amylopectin was found to influence the mobility of water in the pastes to a lesser extent with changes in mean correlation times of approximately 10–15% over 90 days. On retrogradation, amylopectin, Arabic and xanthan gums hindered the formation of solid phase structures. Guar gum evoked an increase in mean correlation times of approximately 40–50% during the prolonged process of changes of the molecular dynamics of water. This indicates continued expansion of the polymeric network. Mean correlation time available from spin–lattice and spin–spin relaxation times can be useful in the analysis of the rotational vibrations of the water molecules in biopolymeric structures.

## 1. Introduction

Starch is a naturally abundant polymer produced by many plants as a means of energy storage. It has been used for years not only in the production of food [1,2,3] but also in other industries [4,5]. In contrast to several other starches, corn starches gelatinize in a specific, more complex manner [6,7,8,9]. Their retrogradation is also unique. The short-term retrogradation of normal [8,10,11,12,13,14] and waxy [15,16,17,18,19] corn starches has been fairly well described. Fisher and Thompson [20] and Liu et al. [21] showed that retrogradation was controlled to the greatest extent by the residual crystalline structures of double helices of amylopectin chains in corn starch. Considerable attention was paid to the possibility of controlling retrogradation of corn starch pastes with selected additives such as galactomannans and citric acid [22], porcine plasma protein hydrolyzates [23,24], various disaccharides [25] and branched limit dextrins [26]. Promising results encouraged us to study the effect of Arabic, guar and xanthan gums on short- and long-term retrogradation of corn starches.

Our former papers presented the results of studies on short- and long-term retrogradation of potato starches of various amylose content [27] and the effects of addition of non-starchy polysaccharide hydrocolloids on the process [28]. The results of ^1^H NMR analyzes of retrogradation in pastes of these starches blended with non-starchy polysaccharide hydrocolloids were presented in our recent paper [29]. The latter documented that short- and long-term retrogradation was promoted by amylose and accelerated with its increasing content in pastes of two normal potato starches with different amylose content and one waxy potato starch. Arabic, guar or xanthan gums retarded retrogradation of starch pastes. The effect of these non-starchy polysaccharide hydrocolloids, particularly upon long-term retrogradation, was a result of changes in molecular properties of water.

In this paper the details of retrogradation over a prolonged storage of two normal and one waxy corn starch are presented. Based on hardening and syneresis of the starch gels, it could be stated that retrogradation was proportional to the concentration of starch and the content of amylose. A molecular analysis of the retrogradation process in corn starches blended with three non-starchy polysaccharide hydrocolloids was also performed. The analysis was based on measuring ^1^H NMR spin–lattice and spin–spin relaxation times in studied pastes as retrogradation progressed within the period of up to 90 days. The obtained data was than interpreted in terms of the changes of water status. Since the relaxation times are macroscopic parameters [30,31,32], mean correlation times that have microscopic character [33,34,35] were employed.

Selection of starch for this study as well as concentration of the pastes and non-starchy hydrocolloids in binary mixtures was based on our recent studies. Pastes of corn starch in the concentration of 5% and so concentrated binary pastes with selected non-starchy hydrocolloids are used in practice as thickeners for controlling the rheological properties and texture of food products [36,37,38,39,40,41,42,43,44].

## 2. Materials and Methods 

### 2.1. Materials 

Normal corn starch (NCS) of 20.48% amylose, Hylon corn starch (HCS) of 49.75% amylose and waxy corn starch (WCS) of 0.75% amylose were purchased from Cargill Sp. z.o.o. (Warsaw, Poland). Arabic (AG), guar (GG) and xanthan (XG) gums were purchased from Sigma Aldrich (St. Louis, MO, USA).

### 2.2. Sample Preparation

Pastes composed of 5 wt % starch or 4.8 wt % starch with 0.2 wt % hydrocolloid were by heating for 30 min at 90 °C with gentle stirring. Resulting hot pastes (0.2 cm^3^) were passed into measurement vials, sealed with parafilm and allowed to cool to room temperature. Thermally equilibrated samples in the measurement viols were then cooled to 5 °C in an ice bath. 

### 2.3. Relaxation Time Measurements

Measurements of relaxation times were taken after 1, 2, 10, 30 and 90 days of storage at 5 °C. The spin–lattice (*T*_1_) and spin–spin (*T*_2_) relaxation times were measured at 15 MHz with PS15T impulse ^1^H NMR spectrometer (ELLAB, Poznań, Poland) equipped with an integral temperature control system. Prior to the experiments, samples placed in the spectrometer were allowed to heat to 20 °C.

The inversion-recovery (π−t−π/2) impulse sequence [45] was applied for taking *T*_1_ relaxation times. Distances between impulses (*t*) were changed within the range from 100 to 1000 ms at the 20 s repetition time. Each time, 32 FID signals and 119 points from each FID signal were collected. 

Calculations of the spin–lattice relaxation time values were performed with CracSpin software (Jagiellonian University, Cracow, Poland) [46] using a “spin grouping” approach. Marquardt′s method of minimization was applied for fitting multiexponential decays. The accuracy of the relaxation parameters was determined and standard deviations were calculated. Time changes of the current value of the FID signal amplitude in the employed frequency of impulses were described by Equation (1):

(1) $$M_{z}\left(t\right)=M_{0}\left(1-2e^{\frac{-t}{T_{1}}}\right)$$

where: *M*_z_(*t*) is the actual magnetization value, *M*_0_ is the equilibrium magnetization value, *t* is the distance between impulses and *T*_1_ is the spin–lattice relaxation time.

Monoexponential magnetization recovery indicated that the system relaxed within one *T*_1_ spin–lattice relaxation time.

Measurements of the *T*_2_ spin–spin relaxation times were taken using the pulse train of the Carr–Purcell–Meiboom–Gill spin echoes (π/2 − TE/2 − (π)_*n*_) [47,48]. The distance between π (TE) impulses was 2 ms and the repetition time was 15 s. The number of spin echoes (*n*) was 100. Tree accumulation signals were employed. 

The calculation of spin–spin relaxation time values was based on adjustment of the values of echo amplitudes to Equation (2) [49,50]:

(2) $$M_{x,y}\left(TE\right)=M_{0}\overset{n}{\underset{i=1}{\sum }}p_{i}e^{\frac{-TE}{T_{2i}}},$$

where: *M*_x,y_ (*TE*) is the echo amplitude; *M*_o_ is the equilibrium amplitude; *TE* is the distance between π impulses and *p*_*i*_ is the fraction of protons relaxing with the *T*_2*i*_ spin–spin relaxation time.

The calculations were performed with dedicated software using a non-linear least-square algorithm. Standard deviation was used to determine the accuracy of the relaxation parameters.

### 2.4. Statistical Analysis

One-way analysis of variance was performed independently for each dependent variable. Post-hoc Tukey HSD (honest significant difference) multiple comparison tests were used to identify statistically homogeneous subsets at α = 0.05. Statistical analysis of the data was performed with Statistica 13 (Dell Software Inc., Round Rock, TX, USA) software.

## 3. Results and Discussion

In Table 1, Table 2, Table 3 and Table 4 the recorded spin–lattice, *T*_1_, and spin–spin, *T*_2_, relaxation times were collected. Their values taken in the two first days of experiments were common for 5% pastes of normal starches (see, for instance, Glöggler et al. [51]). No essential time-dependent changes of these parameters were observed within first 10 days of storage. After a 30 day storage a two-fold decrease in *T*_2_ was observed, whereas values of *T*_1_ declined just after 90 days. Hence, one could assume that up to 30 days water in NCS was bound in a stable manner. Initially, changes were associated with an increase in the role of spin–spin interactions between the protons of the starch chains. Long spin–lattice relaxation times indicated a large quantity of bulk water in the system. A decrease in both relaxation times in pastes of NCS after 90 days pointed to an increase in the amount of bound water. An ordered macroscopic structure was formed in which water molecules had limited mobility. One could deduce that bound water molecules limited mobility.

In the initial storing period, *T*_1_ values for pastes of NCS and WCS were close to one another indicating a similar bulk to bound water ratio in both pastes (Table 1). However, *T*_2_ values in both cases were significantly distinct from each other. In pastes of WCS, *T*_2_ were about twice as high as in pastes of NCS pointing to much lower availability of water in the latter starch and water present in that paste constituted mainly the bulk fraction. Both relaxation times changed only slightly during 30 days of storage. After 90 days, *T*_2_ was shortened as a result of the structural ordering in the pastes. Pastes of HCS showed a single *T*_1_ component and two *T*_2_ components. This finding is atypical for biopolymer gels. Both components of *T*_2_ were determined by water dynamics in the bulk (*T*_2l_) and bound (*T*_2s_) fractions. Two fractions of water characterized by different *T*_2_ are encountered in tissue systems [30,52] and emulsions [53,54]. HCS contained mainly linear polymeric chains. The occurrence of two *T*_2_ components might have been promoted by a relatively low content of starch with respect to the content of water. Hence, in the pastes, protons relaxing with a relatively short time (150–250 ms) constituted a fraction of protons of a low mobility. Likely, they were directly bound to starch. Fraction of protons of *T*_2l_ was characterized by high relaxation times. Thus, these protons belonged to a water fraction, which did not interact with the paste network. Based on the *T*_2l_ criterion this water could be called the free water fraction. Time dependent variations of *T*_1_ and both components of *T*_2_ indicated that significant changes observed in pastes stored for 1 and 2 days might be related to short-time retrogradation. Significant shortening of *T*_1_ accompanied by the extension of *T*_2_ could suggest an impact of the presence of amylopectin in the pastes. Short chains involved in the formation of the network could control the amount of free water without limiting its mobility. On prolonged storage, relaxation parameters changed non-monotonously. These changes resulted from the interactions of water with the polymer. An essential increase in the *T*_1_ values that was observed on the 10th day of storage could be due to reorganization of the water macrostructure evoked by conformational changes of amylase. The p_*i*_ parameter (Equation (2)) that reflects the participation of protons of particular water fractions in the relaxation varied only by 10%. Therefore, its significance in the analysis of the changes of the molecular dynamics of water within 5% starch pastes was limited.

An increase in the turbidity and eventual precipitation that was observed in all the samples indicated that amylose molecules were unstable, which led to their retrogradation. Retrogradation was a result of the shrinkage of amylose molecules caused by a decrease in the kinetic energy and Brownian motion of the polymer and water molecules. This shrinkage promoted intra- and inter-molecular hydrogen bonding between both the hemiacetal oxygen atom and the adjacent OH-6 of the D-glucopyranosyl residues, and the O-6 and OH-2 of D-glycopyranosyl residues of different molecules. Such bonding facilitated the precipitation of the amylose molecules from the aqueous medium.

Table 2, Table 3 and Table 4 contain values of *T*_1_ and *T*_2_ recorded for binary pastes of corn starches with AG, GG and XG, respectively. For binary pastes of HCS invariantly two components of *T*_2_ were observed, which meant that these pastes contained two independent water fractions. Time dependent changes of the relaxation parameters were perturbed by admixed gums but trends of the changes remained the same as in hydrocolloid free pastes. AG limited the content of bulk water fraction while causing the content of bound water fraction to increase. Thus, AG improved water binding in the biopolymer network.

*T*_1_ values for binary pastes of NCS with GG resembled these for the binary NCS + AG pastes. Both gums influenced water binding in the pastes to the same extent (Table 2 and Table 3). However, *T*_2_ in the HCS + GG pastes was shorter than that in the HCS + AG pastes. This observation indicates that water mobility was limited to a greater extent in network that contained GG. *T*_2_ for the WCS pastes reflected the mobility of the bound water fraction. The bulk water fraction in the binary WCS + GG pastes had better opportunity for molecular movements than it had in the WCS + AG pastes.

An admixture of XG to corn starch pastes of NCS and WCS resulted in negligible changes of both relaxation times (Table 4). Thus, on storage, water in both these systems did not change its molecular properties. Binary WCS-XG paste differed in this respect from the hydrocolloid-free WCS paste. Amount of the bulk water fraction compared to the amount of bound water fraction was reduced what was manifested by longer *T*_1_. The water in bound fraction was more mobile whereas the water in bulk fraction faced strong limitations. Hence, one could deduce that XG did not interact with WCS and observed mobility limitations were caused by the hydrocolloid.

**Figure 1:**
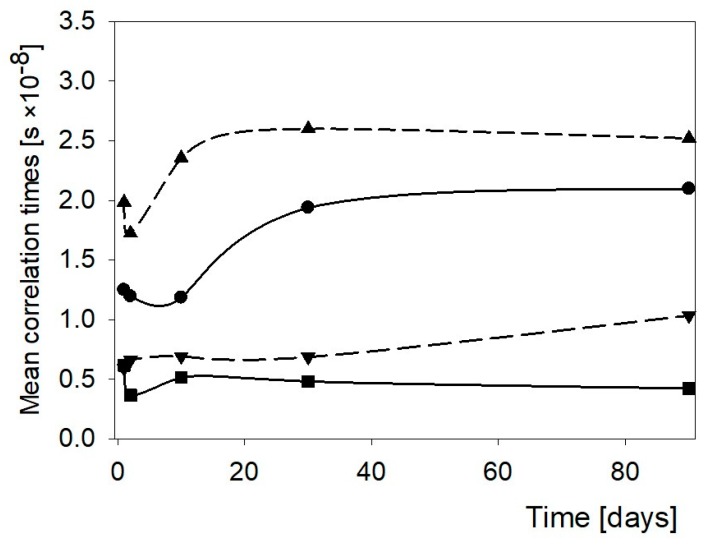
Changes of the mean correlation times in corn starch gels. ● NCS, ▼ WCS, ▲ HCS (s-fraction), and ■ HCS (l-fraction).

Results of the relaxation time measurements showed a path of changing relations between bulk and bound water. Mean correlation time was used to describe the molecular dynamics of water. Time dependent changes of that parameter in starch pastes free of hydrocolloids under study are presented in Figure 1.

As macroscopic parameters, relaxation times reflected mutual relations between bulk and bound water in the system (*T*_1_) and its mobility (*T*_2_). 

Solution of Equations (3) and (4) [32] provided mean correlation time parameter, τ_c,_ describing rotational motions of the water molecules [30,35]. For pure water that parameter reached 10^−12^ s and for ice it decreased to 10^−6^ s [55]. Such a broad range of τ_c_ allows a detailed molecular analysis of pastes and similar systems.

(3) $$\frac{1}{T_{1}}=\frac{6}{20}\frac{\mu_{0}^{2}}{16\pi^{2}}\frac{\gamma^{4}\hbar^{2}}{r_{0}^{6}}\left[\frac{\tau_{c}}{1+{\left(\varpi \tau_{c}\right)}^{2}}+\frac{4\tau_{c}}{1+{\left(2\varpi \tau_{c}\right)}^{2}}\right]$$

(4) $$\frac{1}{T_{2}}=\frac{3}{20}\frac{\mu_{0}^{2}}{16\pi^{2}}\frac{\gamma^{4}\hbar^{2}}{r_{0}^{6}}\left[3\tau_{c}+\frac{5\tau_{c}}{1+{\left(\varpi \tau_{c}\right)}^{2}}+\frac{2\tau_{c}}{1+{\left(2\varpi \tau_{c}\right)}^{2}}\right]$$

As it can be seen in Figure 1, there were two mean correlation times for the HCS paste, that is, one related to bound water, s, and the other to bulk water, l. Changes of τ_c_ over time in pastes of NCS and the s fraction of HCS had the same character. τ_c_ for the s fraction was shorter indicating a more solid state character of this fraction. The l-fraction had a liquid character. Corresponding values of that parameter were lower than these for WCS. In the liquid fraction of HCS system, changes were observed during the first 10 days. Analysis of the changes in τ_c_ for pure corn starch pastes under study revealed that the changes proceeded in three characteristic periods. Initially, within a few days microviscosity of pastes of NCS and HCS declined. In the WCS paste such an effect did not appear. Thus, a conclusion could be drawn that amylose chains had the greatest effect on short-time changes. Within 30 subsequent days molecular dynamics of water in every paste changed the most significantly regardless of the starch variety. They were most remarkable in the paste of NCS and in the s-fraction of the WCS paste. Changes in the l-faction of the latter paste were subtle. On storage longer than 30 days, the network structures stabilized.

**Figure 2:**
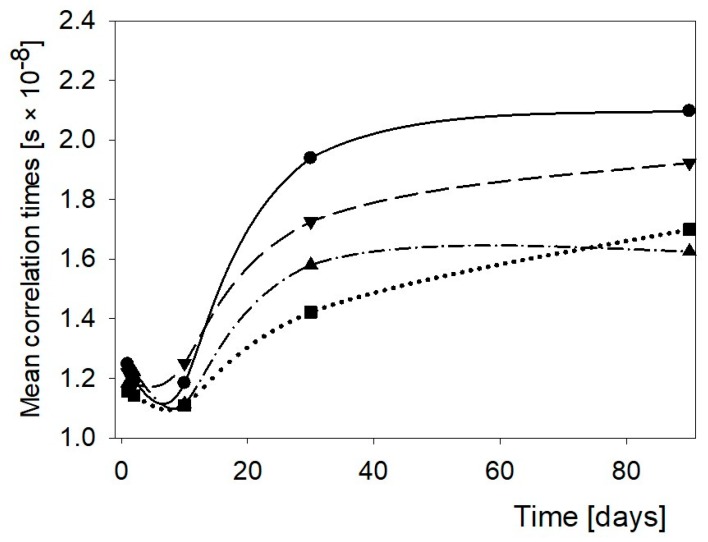
Changes of the mean correlation times of NCS paste and its binary mixtures with hydrocolloids. ●—NCS, ▼—NCS + AG, ■—NCS + GG, and ▲—NCS + XG.

Figure 2 presents changes of mean correlation times in binary pastes of the NCS—hydrocolloid pastes.

Mean correlation times always decreased after admixing a hydrocolloid and that effect was observed within whole storage period. It resulted from repulsing water from the system in which interaction between starch and water was replaced with interactions of starch with hydrocolloid. The course of the time dependent changes in the binary pastes was similar. The minimum τ_c_ was observed within the first 2–10 days of storage. It could be associated with short-time retrogradation of starch in pastes with simultaneous repulsion of water out of the network. After approximately 10 days, τ_c_ rose considerably. Proceeding binding of water in the structure of the polymeric network hindered rotational motions of the water molecules. Admixed hydrocolloids significantly decreased the role of that effect. GG appeared the least efficient in that respect. Thus, the addition of this gum to NCS paste proved the most efficient. However, on prolonged storage, τ_c_ increased in this paste, which suggests that GG bound water even after retrogradation was completed. XG and AG also interacted with starch in the relevant pastes repulsing water from the network but on extended time they had no effect on the molecular dynamics of water. 

**Figure 3:**
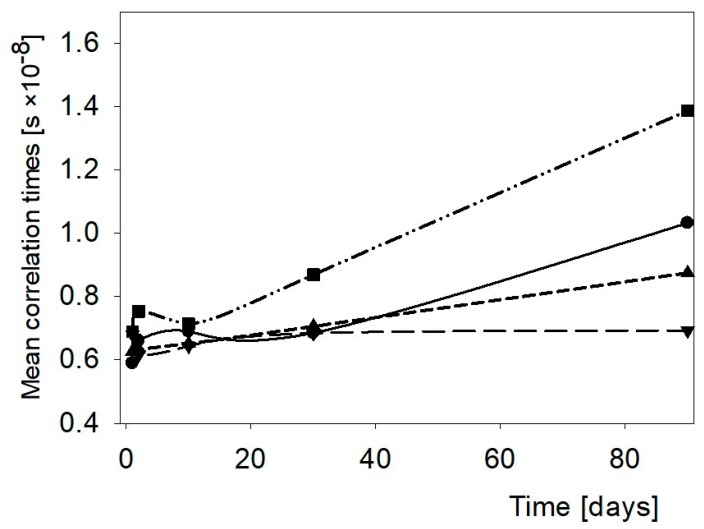
Changes of the mean correlation times in the WCS paste and the WCS binary pastes with hydrocolloids. ●—WCS, ▼—WCS + AG, ▲—WCS + XG, ■—WCS + GG.

Effect of hydrocolloids upon molecular dynamics of water in WCS pastes is presented in Figure 3. Within first 10 days the changes were similar. Among hydrocolloids, GG was the most efficient in inhibiting molecular motions of water. During prolonged storage, τ_c_ rose considerably in this binary paste. Simultaneously, in contrast to the NCS paste, the structure of the network of the WCS paste developed successively which suppressed the mobility of water molecules. The presence of GG intensified formation of the solid state structure. Both of the other hydrocolloids decreased τ_c_ as they provided more freedom for the rotational motions. Likely, both these hydrocolloids hindered retrogradation of amylopectin and AG was particularly efficient in this respect.

**Figure 4:**
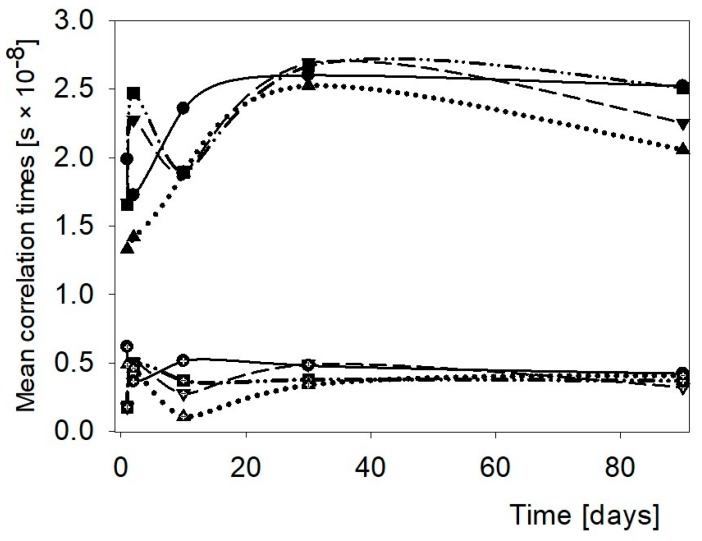
Changes of the mean correlation times in the HCS paste and the HCS binary pastes with hydrocolloids. ●—HCS, ▼—HCS + AG, ▲—HCS + XG, and ■—HCS + GG.

Time dependent changes of water mobility in the binary HCS—hydrocolloid pastes are demonstrated in Figure 4. Effect of hydrocolloid was the most potent within the initial few days. The addition of the hydrocolloids resulted in decreased microviscosity of the fraction of bulk water of the polymeric systems. The effect was the strongest when XG was introduced. After 30 days the effect of hydrocolloids cased. Initially, the water mobility of the bound water fraction in the HCS pastes was hindered by AG and GG. τ_c_ reached certain minima within the first 10 days, which suggests an essential role of both hydrocolloids in the short-term retrogradation of amylose. After approximately 30 days, a further decrease in τ_c_ for all systems was observed. It could point to changes in the amylose network. The polymer–polymer interactions repulsed water out of that network. Only GG disturbed that process. 

## 4. Conclusions

Pastes of starch with high amylose content (approximately 50%) contained two different fractions of water molecules. They were these bound to amylose and amylopectin, respectively. The water molecules of the s-fraction (the fraction of bound water) were characterized by long mean correlation times. These times were an order of magnitude higher than those of the l-fraction. Thus, amylose was found to be responsible for the formation of the polymeric network. The amylopectin fraction (l-fraction) controlled the mobility of water in the pastes to a much lesser extent and it had a minor influence on the formation of network. This conclusion was based on the short mean correlation time values on the one hand and small differences (of approximately 25%) observed between the first and the 90th day of storage on the other.

Normal and waxy corn starches formed compact structures on prolonged storage only. Elevated content of amylose accelerated the formation of such structure. Initially polymer–polymer interactions dominated. They manifested themselves through times of the spin–spin relaxation shortened by 40–60%.

The amount of bound water increased with the amylose content in the system. An increase in *T*_1_ relaxation time was observed from 1401 to 1612 ms for pastes containing 50% to 99% amylose in starch. Hydrocolloids in the pastes shortened mean correlation times within the first ten days of storage. Thus, binary pastes had lower microviscosity than the pastes of pure starches. During retrogradation of amylopectin, Arabic and xanthan gums hindered the formation of solid phase structures. This was indicated by a 20–40% increase of mean correlation time values during 90 days of storage. Guar gum, in contrast, evoked an increase in mean correlation times in a prolonged process of changes of molecular dynamics of water. It was caused by a continued expansion of the polymeric network. The τ_c_ parameter, calculated on the basis basic parallel processes (BPP) equations, allowed the analysis of changes of water mobility in the studied polymeric systems.

## Figures and Tables

**Table 1:** Mean relaxation times of spin–lattice *T*_1_ and spin–spin *T*_2_, in stored corn starch pastes.

Storage Time (days)	NCS		WCS		HCS		
	*T*_1_ (ms)	*T*_2_ (ms)	*T*_1_ (ms)	*T*_2_ (ms)	*T*_1_ (ms)	*T*_2_s * (ms)	*T*_2_l ** (ms)
1	1663 ± 5 ^a^	471 ± 3 ^a^	1612 ± 5 ^d^	935 ± 5 ^a^	1401 ± 4 ^b^	220 ± 1 ^b^	785 ± 7 ^e^
2	1569 ± 4 ^b^	467 ± 5 ^b^	1650 ± 4 ^c^	864 ± 4 ^c^	1257 ± 3 ^d^	239 ± 4 ^a^	966 ± 2 ^b^
10	1557 ± 8 ^b^	468 ± 4 ^b^	1687 ± 8 ^b^	876 ± 6 ^b^	1404 ± 4 ^b^	172 ± 3 ^c^	896 ± 4 ^d^
30	1559 ± 7 ^b^	253 ± 3 ^c^	1697 ± 4 ^a^	874 ± 3 ^b^	1385 ± 4 ^c^	146 ± 2 ^e^	922 ± 9 ^c^
90	1280 ± 6 ^c^	186 ± 3 ^d^	1614 ± 4 ^d^	563 ± 8 ^c^	1411 ± 8 ^a^	156 ± 5 ^d^	1010 ± 2 ^a^

* For bound water fraction. ** For bulk water fraction. Mean values ± SD with different letters in the columns are significantly different at α = 0.05. NCS—normal corn starch; WCS—waxy corn starch; HCS—Hylon corn starch.

**Table 2:** Mean relaxation times of spin–lattice, *T*_1_ and spin–spin, *T*_2_, in stored corn starch—Arabic gum binary pastes.

Storage Time (days)	NCS + AG		WCS + AG		HCS + AG		
	*T*_1_ (ms)	*T*_2_ (ms)	*T*_1_ (ms)	*T*_2_ (ms)	*T*_1_ (ms)	*T*_2_s * (ms)	*T*_2_l ** (ms)
1	1507 ± 4 ^a^	438 ± 4 ^b^	1621 ± 4 ^b^	840 ± 5 ^c^	1296 ± 2 ^b^	257 ± 4 ^a^	1204 ± 4 ^a^
2	1511 ± 2 ^a^	455 ± 5 ^a^	1613 ± 4 ^c^	904 ± 5 ^a^	1256 ± 2 ^c^	162 ± 4 ^c^	808 ± 5 ^e^
10	1483 ± 4 ^b^	419 ± 5 ^c^	1608 ± 5 ^d^	871 ± 4 ^b^	1339 ± 1 ^a^	225 ± 3 ^b^	1138 ± 5 ^b^
30	1458 ± 5 ^c^	277 ± 4 ^d^	1635 ± 2 ^a^	843 ± 5 ^c^	1292 ± 4 ^b^	129 ± 4 ^d^	849 ± 4 ^d^
90	1365 ± 1 ^d^	224 ± 7 ^e^	1549 ± 3 ^e^	790 ± 3 ^d^	1260 ± 2 ^c^	165 ± 5 ^c^	1018 ± 7 ^c^

* For bound water fraction. ** For bulk water fraction. Mean values ±SD with different letters in the columns are significantly different at α = 0.05. NCS + AG—normal corn starch with Arabic gum; WCS + AG—waxy corn starch with Arabic gum; HCS + AG—Hylon corn starch with Arabic gum.

**Table 3:** Mean relaxation times of spin–lattice, *T*_1_ and spin–spin, *T*_2_, in stored corn starches—guar gum binary pastes.

Storage Time (days)	NCS + GG		WCS + GG		HCS + GG		
	*T*_1_ (ms)	*T*_2_ (ms)	*T*_1_ (ms)	*T*_2_ (ms)	*T*_1_ (ms)	*T*_2_s * (ms)	*T*_2_l ** (ms)
1	1522 ± 2 ^a^	470 ± 4 ^a^	1700 ± 3 ^ab^	871 ± 5 ^a^	1228 ± 2 ^c^	246 ± 5 ^a^	1142 ± 4 ^a^
2	1415 ± 2 ^c^	443 ± 5 ^b^	1709 ± 3 ^a^	811 ± 5 ^c^	1194 ± 2 ^d^	136 ± 5 ^d^	778 ± 5 ^d^
10	1379 ± 4 ^d^	446 ± 7 ^b^	1690 ± 4 ^b^	840 ± 7 ^b^	1298 ± 4 ^b^	218 ± 7 ^b^	989 ± 4 ^c^
30	1462 ± 2 ^b^	355 ± 7 ^c^	1666 ± 3 ^c^	692 ± 5 ^d^	1346 ± 3 ^a^	136 ± 8 ^d^	1017 ± 4 ^b^
90	1367 ± 1 ^e^	267 ± 5 ^d^	1596 ± 3 ^d^	499 ± 6 ^e^	1300 ± 3 ^b^	145 ± 5 ^c^	994 ± 2 ^c^

* For bound water fraction. ** For bulk water fraction. Mean values ±SD with different letters in the columns are significantly different at α = 0.05. NCS + AG—normal corn starch with guar gum; WCS + AG—waxy corn starch with guar gum; HCS + AG—Hylon corn starch with guar gum.

**Table 4:** Mean relaxation times of spin-lattice *T*_1_ and spin-spin *T*_2_ in stored corn starches—xanthan gum binary pastes.

Storage Time (days)	NCS + XG		WCS + XG		HCS + XG		
	*T*_1_ (ms)	*T*_2_ (ms)	*T*_1_ (ms)	*T*_2_ (ms)	*T*_1_ (ms)	*T*_2_s * (ms)	*T*_2_l ** (ms)
1	1558 ± 4 ^d^	869 ± 5 ^ab^	1574 ± 2 ^e^	871 ± 4 ^ab^	1362 ± 2 ^b^	358 ± 2 ^a^	497 ± 6 ^c^
2	1587 ± 4 ^c^	839 ± 4 ^a^	1597 ± 2 ^c^	877 ± 5 ^a^	1225 ± 2 ^d^	298 ± 3 ^b^	459 ± 5 ^e^
10	1642 ± 5 ^b^	877 ± 4 ^b^	1612 ± 4 ^b^	865 ± 4 ^b^	1337 ± 1 ^c^	225 ± 2 ^c^	801 ± 5 ^a^
30	1657 ± 4 ^a^	710 ± 6 ^c^	1677 ± 2 ^a^	842 ± 4 ^c^	1388 ± 4 ^a^	153 ± 5 ^e^	617 ± 4 ^b^
90	1542 ± 7 ^cd^	698 ± 5 ^bc^	1582 ± 3 ^d^	653 ± 5 ^d^	1225 ± 4 ^d^	183 ± 2 ^d^	472 ± 5 ^d^

* For bound water fraction. ** For bulk water fraction. Mean values ± SD with different letters in the columns are significantly different at α = 0.05. NCS + AG—normal corn starch with xanthan gum; WCS + AG—waxy corn starch with xanthan gum; HCS + AG—Hylon corn starch with xanthan gum.
